# Cognitive Process in Patients With Obsessive-Compulsive Disorder: A Cross-Sectional Analytic Study

**DOI:** 10.32598/bcn.9.6.448

**Published:** 2018-11-01

**Authors:** Saeid Yazdi-Ravandi, Farshid Shamsaei, Nasrin Matinnia, Jamal Shams, Abbas Moghimbeigi, Ali Ghaleiha, Mohammad Ahmadpanah

**Affiliations:** 1. Behavioral Disorders and Substance Abuse Research Center, Hamadan University of Medical Sciences, Hamadan, Iran.; 2. Department of Nursing, Faculty of Basic Sciences, Hamadan Branch, Islamic Azad University, Hamadan, Iran.; 3. Behavioral Sciences Research Center, Shahid Beheshti University of Medical Sciences, Tehran, Iran.; 4. Modeling of Noncommunicable Disease Research Center, Department of Biostatistics, School of Health, Hamadan University of Medical Sciences, Hamadan, Iran.

**Keywords:** Obsessive Compulsive Disorder, Cognitive process, Information processing

## Abstract

**Introduction:**

In recent studies, deficit in cognitive process has been investigated as one of the etiological hypotheses in a wide range of Obsessive-Compulsive Disorder (OCD). This research aimed to compare cognitive process in patients with OCD and healthy groups.

**Methods:**

In the current cross-sectional analytic study, 43 patients with OCD and 43 healthy individuals matched with gender, age, educational and marital status were selected by convenience sampling method and assessed by Wisconsin Cart Sorting Test (WCST), Paced Auditory Serial Addition Test (PASAT) and Yale-Brown Obsessive Compulsive Scale (Y-BOCS). The obtained data were analyzed with Chi-square, Independent t test, Mann-Whitney U test and Pearson correlation in SPSS version16.

**Results:**

There was no difference between the patients with OCD and the healthy group in demographic characteristics (P>0.05). There was a significant differences between two group on the all subscale of WCST test and PASAT3, PASAT2 tests (P<0.01). These findings indicate that the OCD patient’s performance in cognitive process was significantly worse than the healthy controls.

**Conclusion:**

The findings indicate that individuals with OCD suffer from a deficiency in various aspects of cognitive processes. Therefore, paying attention to these deficiencies can make an important contribution to the treatment of these patients.

## Highlights

- Considerable correlation was observed between Obsessive Compulsive Disorder (OCD) symptoms severity and weak cognitive performances.
- OCD patients’ function in cognitive process was significantly worse than the healthy subjects.
- OCD patients’ performance on Wisconsin Cart Sorting and Paced Auditory Serial Addition tests was worse than healthy subjects

## Plain Language Summary

Obsessive-Compulsive Disorder (OCD) is currently recognized as one of the disabling mental illnesses. It is characterized by recurrent, intrusive, and unwanted thoughts (Obsession), impulses, and images, often associated with compulsive behaviors that are repetitive, ritualized and often time consuming (Compulsion). In recent studies, deficit in cognitive process has been investigated as one of the etiological hypotheses in a wide range of these patients. Out of all cognitive impairments, deficiencies in information processing and executive functions greatly restrain patient’s abilities to preserve, acquire and relearn the required skills for suitable performances. This research aimed to compare cognitive process in patients with OCD and healthy subjects. Fourty-three patients with OCD and 43 healthy individuals matched with gender, age, educational and marital status were investigated by three cognitive processes assessment (Wisconsin Cart Sorting Test [WCST], Paced Auditory Serial Addition Test [PASAT] and Yale-Brown Obsessive Compulsive Scale [Y-BOCS]). The obtained data were showed that the OCD patients’ performance in cognitive process was significantly worse than the healthy controls. Also, there is a considerable correlation between OCD symptoms severity and poor cognitive performance. The findings indicate that individuals with OCD suffer from deficiencies in various aspects of cognitive processes. Therefore, paying attention to these deficiencies can make an important contribution to the treatment of these patients.

## 1. Introduction

Obsessive-Compulsive Disorder (OCD) is currently recognized as one of the disabling mental illnesses. “It is characterized by recurrent, intrusive, and unwanted thoughts (obsession), impulses, and images, often associated with compulsive behaviors that are repetitive, ritualized and often time consuming″ (American Psychiatric Publication, 2013). The usual obsessions include contamination, doubting, ordering/symmetry, aggressive thoughts, religion, and sexual imagery (Benzina, Mallet, Burguière, N’diaye, & Pelissolo, 2016).

Several research studies have reported that patients with OCD have many deficits in cognitive flexibility, set shifting, attention and memory (Tükel et al., 2012; Aydın, Koybasi, Sert, Mete, & Oyekcin, 2014). Although dysfunctions in cognitive abilities are obvious in the daily behavior of OCD patients, fundamental research on neurocognitive dysfunction is still ongoing, and the dimensions of this type of dysfunction are unknown (Chamberlain, Blackwell, Fineberg, Robbins, & Sahakian, 2005). Among all cognitive impairments, deficiencies in information processing and executive functions greatly restrain patient’s abilities to preserve, acquire and relearn the required skills for suitable performances (Keefe, 1995).

Cognitive process dysfunction hypotheses have been used to explain many psychological disorders such as OCD (Penadés, Catalán, Andrés, Salamero, & Gastó, 2005), schizophrenia (Moritz et al., 2002), autism (Tanguay, 2000), and bipolar disorders (Balanzá-Martínez et al., 2010).

Based on different studies, patients with OCD demonstrated specific cognitive deficits on attention, executive and memory functions tasks (Rotge et al., 2008; Bentzina et al., 2016). Patients with OCD have an impaired performance pattern, which is qualitatively like the performance of patients with frontal lobe excisions and sub-cortical pathology. In addition, several research studies reported abnormalities in the cortico-basal ganglion circuits in OCD (Rotge et al., 2008; Benzina et al., 2016).

Converging findings from imaging research implicate abnormalities in frontal-subcortical circuitry in the OCD pathogenesis (Saxena & Rauch, 2000; Lewin et al., 2014). Functional and structural imaging in OCD showed hyperactivity in the limbic, associative cortical area, and related basal ganglia (Menzies et al., 2008). Recent studies recommend that neurobiological abnormalities play a main role in the cognitive dysfunction of these patients (Abramovitch, Abramowitz, & Mittelman, 2013; Benzina et al., 2016).

Many neurocognitive studies suggest abnormalities in the cognitive process of individuals with OCD (Chamberlain et al., 2007; Remijnse et al., 2006; Viswanath, Janardhan Reddy, Kumar, Kandavel, & Chandrashekar, 2009). However, not all studies have found abnormalities and when abnormalities are detected, the results are not always consistent (Moritz, Kloss, & Jelinek, 2010).

Although cognitive processes have been investigated in OCD and promising results have been obtained, sometimes these findings were controversial. Considering the mentioned issues, the identification of various aspects of the cognitive performances of these patients can contribute in better understanding of the existing viewpoints on this disorder. The evaluation of cognitive processes (executive functions and information processing) can be applied in the treatment of these patients. This study aimed to compare the cognitive process in OCD patients and healthy control individuals. The authors hypothesized that OCD patients would perform weaker than healthy control individuals in the executive function and information processing areas.

## 2. Methods

This cross-sectional descriptive-analytical research was conducted in Farshchian-Sina Hospital of Hamadan City, Iran in 2017. This study included 43 patients who met the DSM-IV-TR criteria for OCD (50 patients were interviewed, seven of whom did not meet the study inclusion criteria). The control group consisted of 43 healthy participants who were selected from Farshchian Hospital and Hamadan University of Medical Sciences personnel and students, who matched the patients in terms of gender, age and education.

The participants of the two groups were selected by convenience sampling method. Patients in the current study were included as per the following criteria: 1. Having diagnosed with OCD by a psychiatrist according to the Structured Clinical Interview for DSM-IV Clinical Version (Persian edition); 2. Being 18 to 65 years old; 3. Having at least elementary level of education; 4. Taking a score of at least 16 on the Yale-Brown Obsessive Compulsive Scale (Y-BOCS); and 5. Lacking any current psychiatric disorder except diagnosis of OCD.

Patients were not included in the study if they had 1. A history of or current drug and/or alcohol abuse or dependency (self-report); 2. Any neurologic disease or concomitant general medical condition; 3. Serious head injury history; 4. Intellectual disability or any clinical condition that could affect cognitive performance; 5. Significant medical illness; 6. Electroconvulsive therapy in the last year; 7. Physical disability (e.g. blindness, deafness, speech problems, paralysis, amputation); and 8. Chosen to withdraw from the study.

### 2.1. Clinical measures

The sociodemographic profile included name, age, gender, address, marital status, and education.

#### 2.1.1. Structured Clinical Interview for DSM-IV Disorders

Structured Clinical Interview for DSM-IV Disorders (SCID-I) is a structured interview method that provides information to diagnose Axis-I psychiatric disorders included in DSM (First, Spitzer, Gibbon, & Williams, 1997). In the current study, the Persian version was used. Administration of this test usually takes about 45 to 90 minutes in a single session (First, Spitzer, Gibbon, & Williams, 1996). Reliability and validity of SCID have been reported acceptable in the Iranian clinical population (Sharifi et al., 2009).

#### 2.1.2. Yale-Brown Obsessive-Compulsive Scale

Yale-Brown Obsessive-Compulsive Scale (Y-BOCS) was developed by Goodman and some other researchers to assess the severity of OCD symptoms (Goodman et al., 1989). Y-BOCS includes 10 self-report items. The first 5 questions relate to obsession, and the other 5 questions relate to compulsion. All items have a score ranging from 0 (symptoms free) to 4 (extreme symptoms). The scores were classified as subclinical (0 to 7), mild (8 to 15), moderate (16 to 23), severe (24 to 33), and extreme (34 to 40). Moreover, Rajezi Esfahani, Motaghipour, Kamkari, Zahiredin and Janbozorgi (2012) studied the validity and reliability of the Persian version of Y-BOCS. They claimed that its test-retest reliability coefficients, split-half reliability, and internal consistency ranged between 0.89 and 0.99.

### 2.2. Cognitive process assessment

#### 2.2.1. Wisconsin Cart Sorting Test

Wisconsin Cart Sorting Test (WCST) measures the executive functions such as flexibility, strategic planning, goal-oriented behavior, and organized searching. Assessing the shift depends on the concepts, and forming abstractions are the main purposes of this test (Spreen & Strauss, 1998). Moreover, a computerized version of a 64-card WCST was used. WCST has 64 cards, containing figures in different shapes: Triangle, star, cross and circle (numbered from 1 to 4). The color of these shapes are red, green, yellow and blue. Therefore, each card has a figure (one of the four shapes), a number (1 to 4), and a color (red, green, yellow or blue).

The combination of these choices provides 64 different variations. For scoring, we considered the following: score of perseveration errors (this error arises when the subject, despite receiving feedback from the assessor, is trying to avert the incorrect response or continues sorting based on the former principle or incorrect guess), number of correct and incorrect responses, trials to first complete category, time of completion (second), and the number of completed categories (which identifies correct sorting according to figures, colors, and numbers).

In this test, higher number of completed categories were considered as a better abstractive capability and lower perseverative error, and higher conceptual level response scores indicated less mental rigidity and better cognitive flexibility (Tükel et al., 2012). Naderi (1994) by doing test-retest reported the reliability of the test as 0.85 between Iranian populations.

### 2.3. Information processing assessment

#### 2.3.1. Paced Auditory Serial Addition Test

To assess the rate of attention and information processing, Paced Auditory Serial Addition Test (PASAT) is used as a serial addition task. In this test, 61 single-digit numbers between 1 and 9 are read randomly to the patients in fixed-time intervals in two versions, i.e. in 3 and 2 seconds. In the current study, both versions were administered.

The patient has to sum each number to the one immediately prior to it. For example, if numbers 4 and 7 are presented, the correct answer would be 11. Higher number of correct answers indicate better performance. A maximum of 60 correct responses can be achieved (Sherman, Strauss, & Spellacy, 1997). Some researchers have indicated that PASAT has a high internal consistency, typically ranged between 0.76 and 0.95 (Macleod & Prior, 1996; Sherman et al., 1997).

### 2.4. Statistical analysis

SPSS Version 22.00 was used for all statistical analyses. Kolmogorov-Smirnov was used to test data normality. The difference between the two groups in numerical variables was evaluated by using the Independent samples t test. If the mentioned assumptions were not met, Mann–Whitney U test was used. For categorical variables, χ^2^ test or Fisher exact test was utilized (Cohen, 1988). Statistics for effect size was calculated for each cognitive measure. Furthermore, to investigate the relationship between the cognitive test and Y-BOCS scores in the OCD group, the Pearson correlation was used.

## 3. Results

The participants’ ages ranged between 19 and 62 years with the Mean±SD age of 35.12±11.00 years in the OCD group, and 32.35±8.81 years in the healthy group. Table 1 presents demographic variables of the two groups and the scores of obsession, compulsion, and total score of Y-BOCS in the OCD group. There was no significant difference in gender, age, and educational and marital status between the two groups based on Chi-square and Fisher exact tests.

**Table 1:** Clinical and demographic characteristics of the study participants

**Variables**		**Groups**			

		**OCD (n=43)**	**Healthy (n=43)**	**Total (N=86)**	**Sig.**
Gender	Male	14(32.6)	20(46.5)	34(39.5)	X^2^=1.75
	Female	29(67.4)	23(53.5)	52(60.5)	P=0.186
Marital status	Single	17(39.5)	20(46.5)	37(43)	X^2^=0.427
	Married	26(60.5)	23(53.5)	49(57)	P=0.514
Education degree^*^	Under diploma	10(23.2)	8(18.6)	18(20.9)	P=0.237
	Diploma	18(41.9)	18(41.9)	36(41.9)	
	College degree	15(34.9)	17(39.5)	32(37.2)	
Age, y		35.12±11.0	32.35±8.81	33.73±10.00	t=1.287 P=0.201
Y-BOCS	Obsession	11.13±2.42			
	Compulsion	10.02±2.67			
	Total score	21.13±4.87			

* Fisher exact test. The data are presented as No. (%) or Mean±SD.

Table 2 presents all the descriptive indices of WSCT in two groups. As it can be seen, compared to healthy control individuals, patients with OCD performed worse in all WCST subtest scores. For example, the Mean±SD of the perseverative error and category were respectively 12.93±7.18 and 2.05±1.44 in the OCD group and 4.95±4.07 and 4.07±1.56 in the control group. In addition to these indicators, in this study, the time spent on the test was also studied as an indicator. As shown in Table 2, OCD patients spent more time to do the test tasks.

**Table 2:** WSCT test results

**WSCT**	**Groups**	**Mean**	**SD**	**Max**	**Min**
Perseverative error	OCD	12.93	7.18	29	2
	Healthy	4.95	4.07	15	0
Categories	OCD	2.05	1.44	6	0
	Healthy	4.07	1.56	6	0
Correct response	OCD	26.72	8.45	42	14
	Healthy	36.98	7.21	48	17
Error response	OCD	33.14	8.74	46	13
	Healthy	20.63	5.21	43	4
Trials to first complete category	OCD	25.19	17.46	60	6
	Healthy	15.49	11.59	51	6
Conceptual response	OCD	2.16	2.65	6	0
	Healthy	5.28	1.81	6	0
Time of completion^*^	OCD	274.19	77.22	451	135
	Healthy	198.98	49.62	320	100

* Second

Independent t test results indicate that the performance of patients with OCD was significantly worse than the healthy control individuals with regard to the perseverative error (P<0.001, Cohen’s d=1.367), category (P<0.001, Cohen’s d=1.365), correct response (P<0.001, Cohen’s d=1.306), error response (P<0.001, Cohen’s d=1.738), trials to first complete category (P=0.003, Cohen’s d=0.654), and time of completion (P<0.001, Cohen’s d=0.115). In addition, in the subscale of the conceptual level responses, the Mann-Whitney U test results indicate a lower performance by OCD individuals (P<0.001, Cohen’s d=1.374). According to Table 3, large effect sizes (1.115 to 1.738) were responsible for the differences between the OCD and the healthy groups, expect trials to first complete category that had medium effect sizes.

**Table 3:** Comparison of WSCT test results between patients with OCD and healthy control individuals

**WSCT**	**T**	**df**	**Effect Size**	**P**	**95% CI**	

					**Lower**	**Upper**
Perseverative error	84	6.334	1.367	<0.001	5.472	10.481
Categories	84	−6.226	1.365	<0.001	−2.669	−1.377
Correct response	84	−6.049	1.306	<0.001	−13.627	−6.884
Error response	84	6.474	1.738	<0.001	8.668	16.355
Trials to first complete category	84	3.033	0.654	0.003	3.340	16.055
Time of completion	84	5.373	1.115	<0.001	47.372	103.046
Conceptual response^*^	-	z: −5.003	1.374	<0.001	-	-

* Mann-Whitney U test

In order to investigate information processing, PASAT3″ and PASAT2″ were conducted. According to Table 4, the Mean±SD of incorrect responses of the OCD group in the PASAT3″ and PASAT2″ test were respectively 16.88±11.14 and 19.26±12.66, indicating that participants of the healthy control group had a better performance in this test. Table 4 presents a significant difference in information processing between the two groups for PASAT3″ (P<0.001, Cohen’s d=0.784) and PASAT2″ (P<0.003, Cohen’s d=0.662). Cohen’s d effect sizes between the two groups were medium.

**Table 4:** Comparison of PASAT test results between patients with OCD and healthy control individuals

**Variable**	**Groups**	**Mean±SD**	**Max**	**Min**	**T**	**df**	**Effect Size**	**P**	**95% CI**	

									**Lower**	**Upper**
PASAT3″	OCD	16.88±11.14	42	3	3.642	84	0.784	<0.001	3.558	12.117
	Healthy	9.05±8.66	42	0						
PASAT2″	OCD	19.26±12.66	45	2	3.069	84	0.662	0.003	2.586	12.111
	Healthy	11.91±9.28	43	1						

As observed in Table 5, there was a considerable correlation between obsession, compulsion, and total score of Y-BOCS with the number of completed category (P<0.05), correct responses (P<0.05), error responses (P<0.05) and conceptual level responses (P<0.01) of WCST. Our findings suggest that the correlation between time of completion with obsession and total score was significant (P<0.05). Moreover, correlation between PASAT3″ and PASAT2″ with obsession subscale of Y-BOCS was significant.

**Table 5:** Correlation of Y-BOCS scores with WCST and PASAT test variables in OCD group

**WSCT**	**Obsession**	**Compulsion**	**Total Score**
Perseverative error	0.141	0.097	0.123
Category	−0.368^*^	−0.461^*^	−0.437^*^
Correct response	−0.318^*^	−0.354^*^	−0.353^*^
Error response	−0.317^*^	−0.355^*^	−0.352^*^
Trials to first complete category	0.097	0.227	0.173
Time of completion	0.364^*^	0.285	0.338^*^
Conceptual response	−0.466^**^	−0.470^**^	−0.490^**^
PASAT3″	0.324^*^	0.193	0.267
PASAT2″	0.303^*^	0.144	0.230

* P≤0.05;

** P≤0.01

## 4. Discussion

The main findings in our investigation revealed a significant difference in information processing and executive function in OCD individuals compared to the healthy control individuals. In the present study, an impairment in cognitive processing was found with the WCST test in patients with OCD. Performance of OCD patients in the present study was weaker than healthy control individuals. They showed more preservative error, error responses, trial to first complete category, time of completion; and fewer categories, correct response, conceptual level response.

This observation agrees with many studies that have reported deficits in the performances of OCD patients in WCST (Penadés et al., 2005; De Geus, Denys, Sitskoorn, & Westenberg, 2007; Ghassemzadeh et al., 2012; Aydın et al., 2014). It suggests that OCD patients have a lower cognitive flexibility/set shifting, are more prone to distraction, and inadequate in abstraction ability (Tükel et al., 2012). Since the cognitive flexibility/set shifting is required to understand abstractive principles, it is difficult for OCD patients to hypothesize and assess it, so it seems that they usually have doubt about everything (Saremi, Shariat, Nazari, & Dolatshahi, 2017).

It is rational to clarify inflexible and persistent thoughts and behaviors of the executive function concept in OCD patients (Saremi et al., 2017). Cognitive deficits were found in patients with OCD, are associated with pre-frontal-striatal dysfunction (Greisberg & McKay, 2003; Lacerda et al., 2003). To be more specific, the brain structures that are mostly related to OCD disorders are the basal ganglia structures and orbitofrontal cortex (De Geus et al., 2007). Perseverative behavior is the most prominent feature of the executive/frontal dysfunction (De Geus et al., 2007). The deficits detected in the present patients fit this profile.

The patients with OCD showed a lower number of completed category and conceptual level responses compared with healthy control individuals, and this illustrates a weaker concept formation. Previous studies have also concluded the same results (Rao, Reddy, Kumar, Kandavel, & Chandrashekar, 2008). However, some research studies report that the WCST score of these patients and the healthy individuals do not differ significantly (Deckersbach et al., 2000; Moritz et al., 2002). This difference in previous research can be due to factors such as patient’s clinical situation, small sample size, intelligence quotient, and medication effects. Another explanation for the present findings on the WCST can be attributed to the attention system dysfunction (Greve, Williams, Haas, Littell, & Reinoso, 1996; Greve, Ingram, & Bianchini, 1998).

Regarding the PASAT, the authors found information processing deficits in patients with OCD in comparison with the healthy control individuals. Patients with OCD often report sensory intolerances, which may lead to important functional impairments. There have been only a few OCD investigations on the stimulus elements’ encoding process, such as attention (Foa & McNally, 1986; Lavy, Van Oppen, & Van Den Hout, 1994). Cognitive theories suggest that OCD should similarly feature abnormal attentional processing toward concern-related materials (Rachman, 1997; Tata, Leibowitz, Prunty, Cameron, & Pickering, 1996; Morein-Zamir et al., 2013).

This deficiency could be related to anterior cingulate cortex dysfunction, a region involved in conflict monitoring in attention functions and information processing (Devinsky, Morrell, & Vogt, 1995; Van Veen & Carter, 2002). Furthermore, obtaining the effect size of Cohen’s d could lead to more reliable and deeper conception about the cases. Based on Cohen’s (1988) definition of effect size, the results of the current study demonstrated a large effect size in all subscales of WCST and medium effect in PASAT3″ and PASAT2″. Effect size values, in accordance with the significance level (P), were evaluated for all cognitive variables, which indicate significant differences between OCD patients and healthy groups. These results showed that healthy control individuals performed better than patients with OCD. Prior meta-analytical research studies presented different effect sizes of various cognitive process subdomains (Abramovitch et al., 2013; Shin, Lee, Kim, & Kwon, 2014; Snyder, Kaiser, Warren, & Heller, 2015).

Our findings showed a considerable negative relation between obsession, compulsion, and total Y-BOCS score with most subscales of the WCST test. PASAT3″ and PASAT2″ scores correlated significantly with obsession. Inconsistent with our result, several research findings have reported no significant correlation between the severity of OCD symptoms and performance in cognitive tests (Saremi et al., 2017; Bédard, Joyal, Godbout, & Chantal, 2009). Also, other studies reported a significant relation between the cognitive process and severity of OCD symptoms (Nedeljkovic et al., 2009; Abramovitch, Dar, Schweiger, & Hermesh, 2011). In fact, the severity of OCD symptoms could probably reduce the ability of these patients in their cognitive processes.

In conclusion, this findings indicate that individuals with OCD suffer from a deficiency in various aspects of the cognitive process such as memory, shifting attention, flexibility, perseveration, and information processing, and they perform more poorly compared to normal individuals. In the current study, some aspects of the cognitive process were reviewed, too. These findings have significance because they suggest that OCD could be considered as a dysfunction of the cognitive ability. Therefore, paying attention to these deficiencies can seriously contribute to the treatment of these patients. Further studies are recommended to further explore the cognitive impairments in patients with OCD.

The current study had some limitations that should be considered. Our participants’ medications may have influenced test performance. The sample size was relatively small in both groups. We used a relatively limited number of cognitive measures that may not have assessed all aspects of the cognitive process, and definitely missed some aspects of cognitive performance that may be impaired in these patients. Future studies should recruit larger samples and employ more comprehensive neuropsychological tests.

## Ethical Considerations

### Compliance with ethical guidelines

The research was approved by the Local Ethics Committee of Hamadan University of Medical Sciences. Prior to participation in the study, an informed written consent was taken from all participants.
